# Influence of *Piper betle L.* extract on umbilical cord cells *in vitro* and potential treating cutaneous wound

**DOI:** 10.1016/j.heliyon.2021.e06248

**Published:** 2021-03-08

**Authors:** Cẩm Hà Chế Thị, Hữu Đạt Nguyễn, Duy Minh Lê Hoàng

**Affiliations:** a Hue University of Sciences, Viet Nam; b Hue University of Medicine and Pharmacy, Viet Nam

**Keywords:** *Piper betle L.*, The extract, Fibroblast cell, Mesenchymal stem cells

## Abstract

This research aimed to test the effects of *Piper betle* L. from Vietnam on fibroblasts and UC-derived mesenchymal stem cells (UC-MSCs) from human umbilical cord (UC) on the scratch assay. We tested the extract at different concentrations and then assessed the level of expression of the factors involved in the inflammatory process on fibroblasts including IL-33, VCAM, CD248 by assay real time qPCR. At the concentrations of 0.025 μL/mL and 0.03 μL/mL, the extracts positively affected fibroblast proliferation and UC-MSCs. By contrast, the concentration of 0.058 μL/mL, the extract was toxic to UC-MSCs and fibroblast cell lines, the cells were no longer able to survive. qPCR results show that *Piper betle* L. extract has the ability to reduce the expression levels of IL-33 (50.8%), VCAM (32.1%), CD248 (46.13%) which trigger inflammatory processes, thereby reducing cellular stress and promoting the process of healing scratches.

Our study revealed the proliferation capabilities of *Piper betle* L. extract from Vietnam In vitro. Hence, *Piper betle* L. could be recommended as a potential source of wound healing agents.

## 1 Introduction

The skin is the organ which covering the body and plays an important role as a physical barrier that protects the body against negative external influences. When this protection is weakened, pathogens have such a straightforward approach to entering the body, probably resulting in infection [1]. The wound healing process is a combination of several successive and overlapping stages: inflammatory response, cell proliferation, and tissue regeneration [2]. During proliferation, fibroblasts play a very important role in supporting wound healing. At the end of the inflammatory phase and the onset of proliferation (after 24–48 h of injury), the first fibroblasts appear at the site of injury, then they infiltrate and degrade the fibrin clot. Fibroblasts are converted to myofibroblasts to create a delicate balance in epithelial regeneration that promotes fibroblast proliferation and migration, accelerates wound retraction [2,3,4]. However, accelerating wound withdrawal can be interrupted for a variety of reasons, activated/damaged fibroblasts in healing tissues or inflammations were characterized by increased expression of IL-33, VCAM-1, and CD248 [5]. The cytokines interleukin 33 (IL-33) is an alarming agent in early inflammatory disease and it is important in the balance between recovery and deterioration in tissue repair [6], and VCAM is molecular has the function of cell adhesion and CD248 is a transmembrane receptor with ligand collagen 1 which is regulated by the inflammation process [7]. The continuous activation of fibroblasts has been considered by the authors to be an essential mechanism determining the development of chronic inflammation, which in turn determines the duration of wound recovery.

Mesenchymal stem cells (MSCs) have been developed as effective methods to improve skin wound healing [8,9]. advantageously, it is necessary to have an abundant source of stem cells to ensure the efficiency of regenerative medicine applications and invasive processes to ensure donor protection [10,11]. A desirable source of transplantable stem cells for wound healing, the human umbilical cord (UC) has many distinct advantages, namely that there is no danger to donors, it is readily available, and there are several distinct advantages of no donor risk, quick accessibility, and a low incidence of graft-versus-host disease. Studies have documented that human UC-derived mesenchymal stem cells (UC-MSCs) can facilitate the spread and migration of skin cells, angiogenesis, and acute wound in diabetic or burn wound models in animals, indicating that UC-MSC therapy is a promising path to wound healing or in conjunction with biological materials [9,10].

*Piper betle* L. is cultivated widely in most of the humid tropical climate of Southeast Asia, including Vietnam. Their leaves are used as a folk medicine because of their medicinal properties. They have a major stimulation effect on pancreatic lipase function, promote digestion, help prevent pulmonary disease, and help treat a variety of diseases including boils, abscesses, conjunctivitis, and constipation [11]. *Piper betle* L. extracts contain bio-compounds that have been shown to have positive effects in the treatment of diabetes, conjunctivitis, mastitis, burns, sores, as well as having antibacterial, anti-inflammatory, antioxidant and anti-cancer properties [12,13,14,15,16,17]. Other studies have reported that the *Piper betle* L. extract also shows potential in wound healing and anti-aging treatment [14,18]. However, scientists are continuing to explore the mechanisms involved, especially their role in stimulating the proliferation of cell lines in the treatment of open wounds, which are still poorly understood.

Current medications have shown good efficacy against antisepsis in open wounds but cause side effects. Therefore, research and the creation of medicinal origins to replace and assist in wound treatment and wound pathology are necessary. Therefore, our study was conducted to study the effect of betle leaf extract and stimulate proliferation of fibroblast cells in humans.

## 2 Materials and methods

### 2.1 Material

*Piper betle* L. was collected in Hue city, Thua Thien Hue province. The sample was tested and classified by the Plant Subdepartment, Department of Biology, University of Science - Hue University.

### 2.2 Preparation of *Piper betle* L. extract

500g fresh betle leaves were cleaned and washed throughly with water and rinsed with distilled water. Fresh leaves were washed and dried at room temperature.The sample was ground and places in an incubator with 70% ethylic alcohol in a ratio of 1:10 and incubated for 5 h at a temperature of 50 °C. The extract was filtered after incubation, and the solvent was extracted at a temperature of 70 °C to 5% by a vacuum rotary evaporator, a *Piper Betle* L. Stored at 4 °C, the extract was collected.

### 2.3 Testing the composition of the extract

*Piper betle* L. extract (3 ml) after collection will be taken to the Drug, cosmetic and food quality control center of Thua Thien Hue province (HueQC). The extract is checked for composition by Gas Chromatography-Mass Spectrometry system (GC-MS).

### 2.4 Sample collection and group allocation

Samples were collected under sterile conditions at HUE Central hospital and stored in airtight in Dulbecco's modified Eagle medium (DMEM)/F-12 (Gibco; Thermo Fisher Scientific, Inc.) inside of an ice chest. The sample would be rejected if the donor is positive for viral hepatitis, syphilis, and HIV.

### 2.5 Isolated fibroblasts from umbilical cord

Total placenta tissue cells were rinsed three times with sterile phosphate-buffered saline (PBS). The gross surface of the umbilical cord lining membrane (UCLM) near the maternal side was scraped using medical instruments and washed in PBS. A total of 2g UCLM tissue was mechanically fragmented into 1 cm^2^. Tissue fragments were subsequently cultured at a density of 5 × 10^4^ cells/ml in 6-well plates with DMEM/F12 and 10% fetal bovine serum (FBS) and put at 37 °C, 5% CO2, in an incubator. Twice a week, the medium was substituted, and morphology under the inverted optical microscope was examined (Olympus Corporation).

### 2.6 Isolation UC-MSCs from Wharton's jelly

The Wharton's jelly was rinsed with PBS three times and physically separated into 1 cm^2^ part. They were then cultured in DMEM and 10% FBS, 1% penicillin, and 1% streptomycin and put in an incubator at 37 °C, 5% CO_2_. The medium was replaced twice a week and morphology under the inverted optical microscope was examined.

### 2.7 Determination of growth curves and population

The first and third generation of UC-MSCs and fibroblast were cultured in 6-well plates (1×10^4^ cells/ml) at 37 °C, 5% CO_2_, then incubated with trypsin. Three wells were randomly chosen and incubated with trypsin for 3 min and counted by using the automated count cell Countess, periodically three times for a period of 7 days, and each well is tracked.

### 2.8 Colony formation assay

After 7 days of culture, the cell sample was fixed with 4% paraformaldehyde for 5 min, then the sample was colored with 0.1% crystals violet for 15 min. Rinsed with purified water three times, the population of cells was counted under a reverse optical microscope.

### 2.9 Immunophenotype analysis

Flow cytometric analysis was conducted using an automatic counter cell Countess (Invitrogen; Thermo Fisher Scientific, Inc.). 2×10^4^ cells of the third generation of UC-MSCs and fibroblasts have been stained with phycoerythrin (PE) and FITC-conjugated monoclonal antibodies for 10 min at 37 °C. Cell surface markers CD34, CD45, CD73, CD90, and CD105 for UC-MSCs and CD34, CD45, CD90 and α-SMA for fibroblast.

### 2.10 Testing the effect of *Piper betle* L. extract on fibroblasts

Fibroblasts were cultured in primary medium in DMEM/F12 + 1% penicillin, 37 °C, 5% CO2. When the cell density reached 70%–80%, transferred the subculture to a 6-well plate. *Piper betle* L. extract was added at different concentrations to the culture medium. It was tested every 3 h for 48 h.

### 2.11 Assessing the ability of the *Piper betle* L. extract to heal scratches on fibroblasts and UC-MSCs

During the secondary culture, when the cell population was relatively homogeneous and the density reached 80–90%. Scratch with a width of 800 μm was created on the cell surface by a sterile pipette tip. The cell was cleaned with PBS to eliminate the suspended cells. Supplement DMEM/F12 + 10% FBS culture medium and *Piper betle* L. extract at different dilution concentrations, cell culture at 37 °C, CO2 5% and monitored every 3 h for 48 h. The experiment was repeated 3 times.

### 2.12 RNA extraction, cDNA synthesis and real time qPCR

After addition of the extract, the expression level of elements on fibroblasts was checked. RNA was isolated from both untreated and treated cells using InviTrap® Spin Universal RNA Mini Kit (STRATEC Biomedical AG, Berlin) as directed by the manufacturer. RNA concentration and purity were measured using a spectrophotometer (Nanodrop 2000, Thermofisher). 100ng of RNA has been translated to cDNA using a High Capacity cDNA reverse transcription kit (Thermofisher). Using the RNase free water to dilute cDNA. qPCR was performed using PowerUp Sybr Green Mastermix (Thermofisher) and 1μl of cDNA was used with forward and reverse primers. Each sample was run in duplicate and standardized to GAPDH endogenous power. Data reflects fold modification from untreated cells (2^−ΔΔCT^).

Primers were designed online using primer-BLAST (www.ncbi.nlm.nih.gov/tools/primer-blast/) and synthesized by PHUSA Biochem Company, Viet Nam.

GAPDH ((f) 5′- ACTAGGCGCTCACTGTTCTCTC-3′
(r) 5′- CCCAATACGACCAAATCCGTTGA-3′)
IL-33 ((f) 5′- AAGCAAAGCCTTGTGTTTCAAGC -3′
(r) 5′- TGTCTTTTGTGCTTTCTACCTGTTT-3′)
CD248 ((f) 5′- CTGCTACGCTCTCTTCCCAC-3′
(r) 5′- CTCCACACATTCGTGTTCGC-3′),
V-CAM ((f) 5′- GGACCACATCTACGCTGACAA-3′
(r) 5′- AACAGTAAATGGTTTCTCTTGAACA-3′)

### 2.13 Statistical analysis

In this study, all experiments were repeated three times and the collected data were analyzed using ANOVA statistical software, with significance level P < 0.05.

## 3 Result

### 3.1 Component test results

The *Piper betle* L. traditional medicine has been used in Vietnam for hundreds, the aim of which is to reveal the potential impact of such a plant on the production of therapeutically active herb therapies of diseases such as flatulence, boils, abscesses, bronchitis, heartburn, and eadache. GC-MS method has identified 16 compounds in *Piper betle* L. extracts. The chemical composition of the extract including carbohydrates, tri-terpenoids, steroids, alkaloids, amino acids, tannins, and essential oils are similar to the results of Alam *et al.* (2015). When his researches evaluated the antioxidant and antitumor effects, and increase in the lifespan of EAC-bearing mice in response to *Piper betle* L. leaf extract was found [18]. The phenolic compounds are mainly found in fresh betel leaf and are resistant to nitrification, antioxidant, antiplatelet, anti-thrombotic effects and inhibits cancer [19]. Indeed, Euganol is a phenolic compound found in *Piper betle* L. extract, has been shown to have antibacterial, antifungal, antioxidant, anti-inflammatory and inhibitory effects on lung and ovarian cancer cells [20,21,22]. Interestingly, the study by Madhumita M *et al.* (2019) Analysis of mass spectrometry by gas chromatography (GC - MS) in essential oil (EO) of betle leaf showed that thirty-three and thirty volatile compounds, comprising 98.41% and 97.34 % of the configured freshly and preserved leaf EO, have a wide variety of biological activities and industrial uses, respectively. Eugenol was proved to be extremely abundant in both EOs with varying percentages and one of the components that plays a crucial role in resistance to *Mycobacterium smegmatis, Staphylococcus aureus,* and *Pseudomonas aeruginosa* types [23]. Or Phytol [24] has the ability to modulate NOX2 expression, a major factor in cellular oxidative stress.

**Figure 1:**
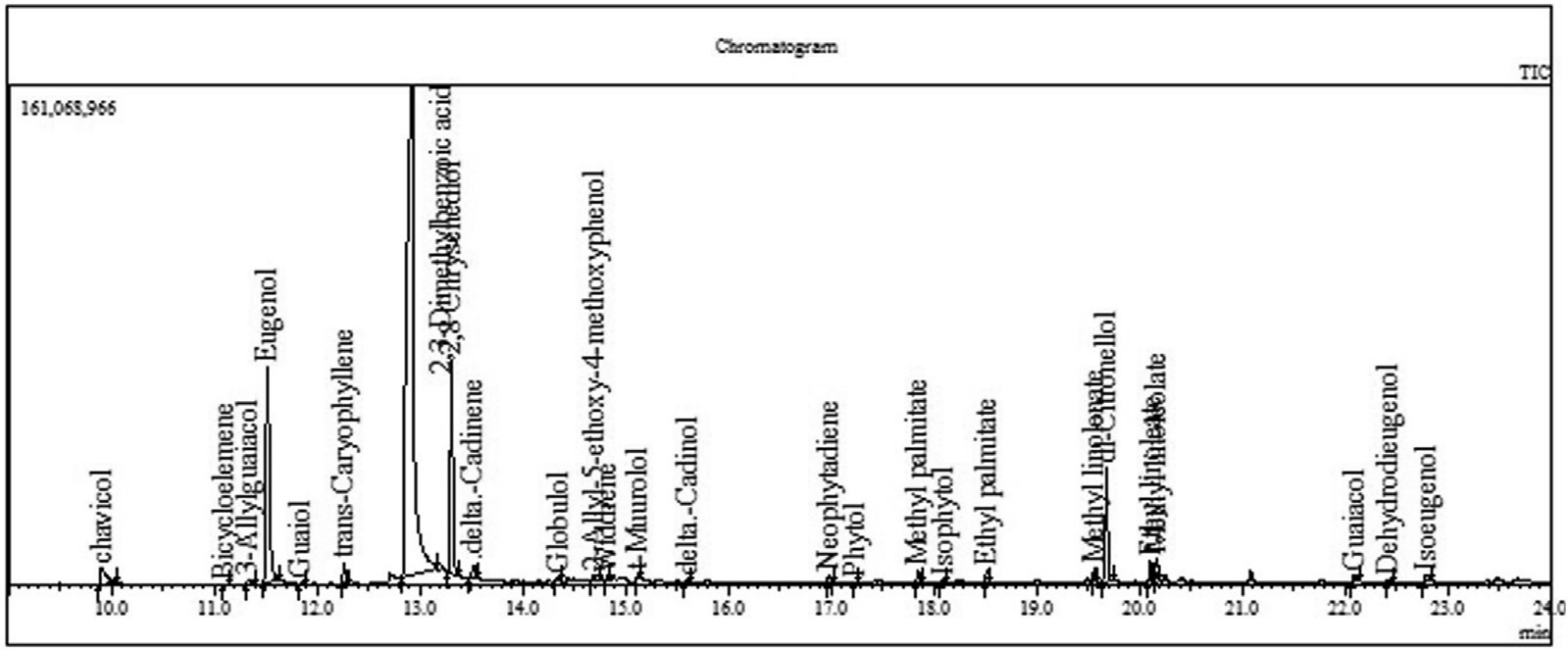
GCMS results.

This study investigates the ability of *Piper betle* extract to stimulate the proliferation of cell lines through the process of scratching in vitro, to provide scientific data on the activities contained in betle leaves, aiming at products with natural origin pharmaceuticals in the treatment of open wound (see Figure 1).

### 3.2 Isolated culture of UC-MSCs and fibroblast

**Figure 2:**
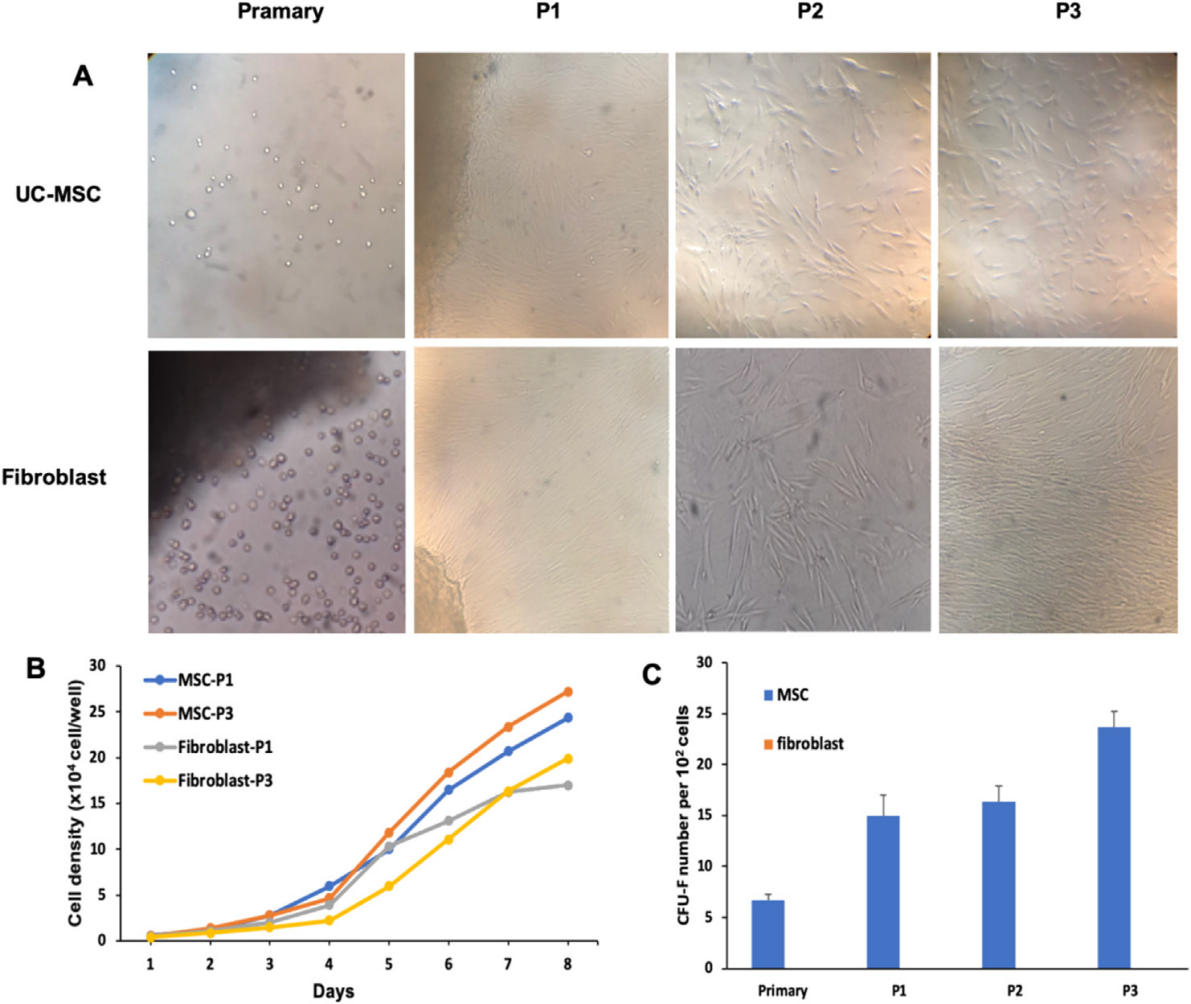
Isolated culture of UC-MSCs and fibroblast. (A) Morphology of UC-MSCs and fibroblast in the primary, first, second and third generation, respectively, (B) Growth curves, (C)colony-forming abilities of UC-MSCs and fibroblast.

Analysis findings the morphology of the two types of cells showed that Wharton's jelly from umbilical cord produced UC-MSCs, and the umbilical cord lining membrane produced fibroblast, a spiral growth structure similar to fibroblast-like cells. The proliferative rate of MSCs was used to test cell growth. The rise curves of the two types of MSCs had an alike pattern. Over the same time span, the number of UC-MSCs in the first and third-generation was higher than the number of fibroblasts (Figure 2A, B).

### 3.3 UC-MSCs exhibit colony forming efficiency

Effective of colony-forming in two groups of MSCs was tested after culture (Figure 2C). Only UC-MSCs exhibit colony-forming efficiency in fimary (6,67 ± 0.57), first (15 ± 2), second (16,3 ± 1,5), and third (23,6 ± 1,5) generation. In contrast, Fibroblast was not formed colony.

### 3.4 Marker expression on UC-MSCs and fibroblast

**Figure 3:**
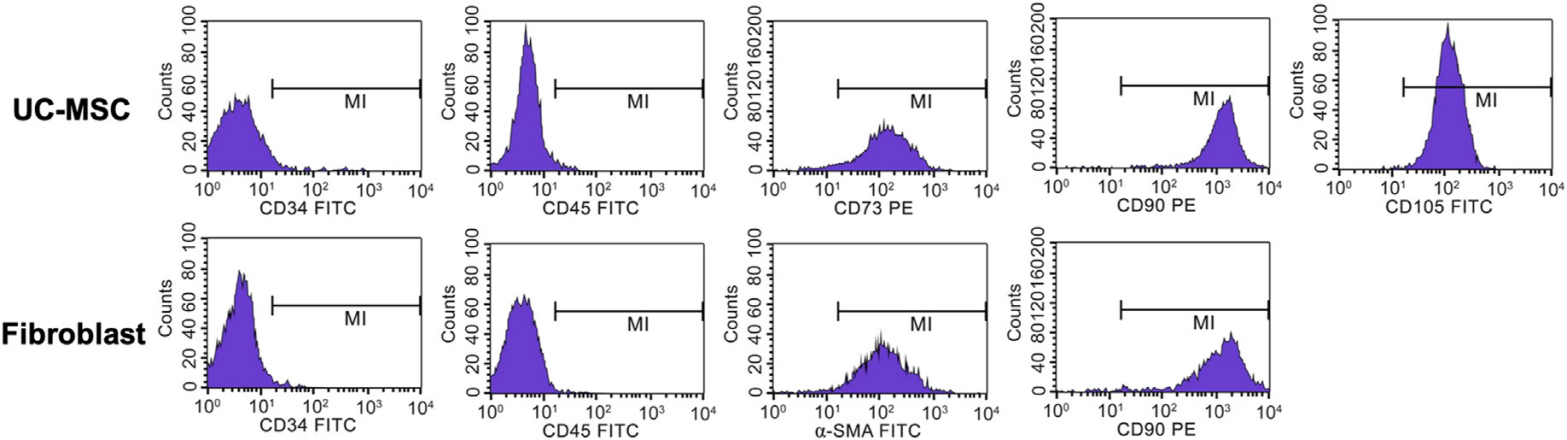
Surface markers of UC-MSCs and fibroblast.

UC-MSCs overexpress the cell-surface markers typical of MSCs CD73, CD90, and CD105 (>95%). In contrast, they exhibit very low levels of CD45 and CD34 (<2%), which are known as characteristic cell surface markers of hematopoietic stem cells. Likewise, fibroblasts clearly expressed the CD90 and α-SMA cell surface markers (>95%) and didn't present CD45 and CD34 (<2%) (Figure 3).

### 3.5 The effect of *Piper betle* L. extract on UC-MSCs and fibroblasts

**Figure 4:**
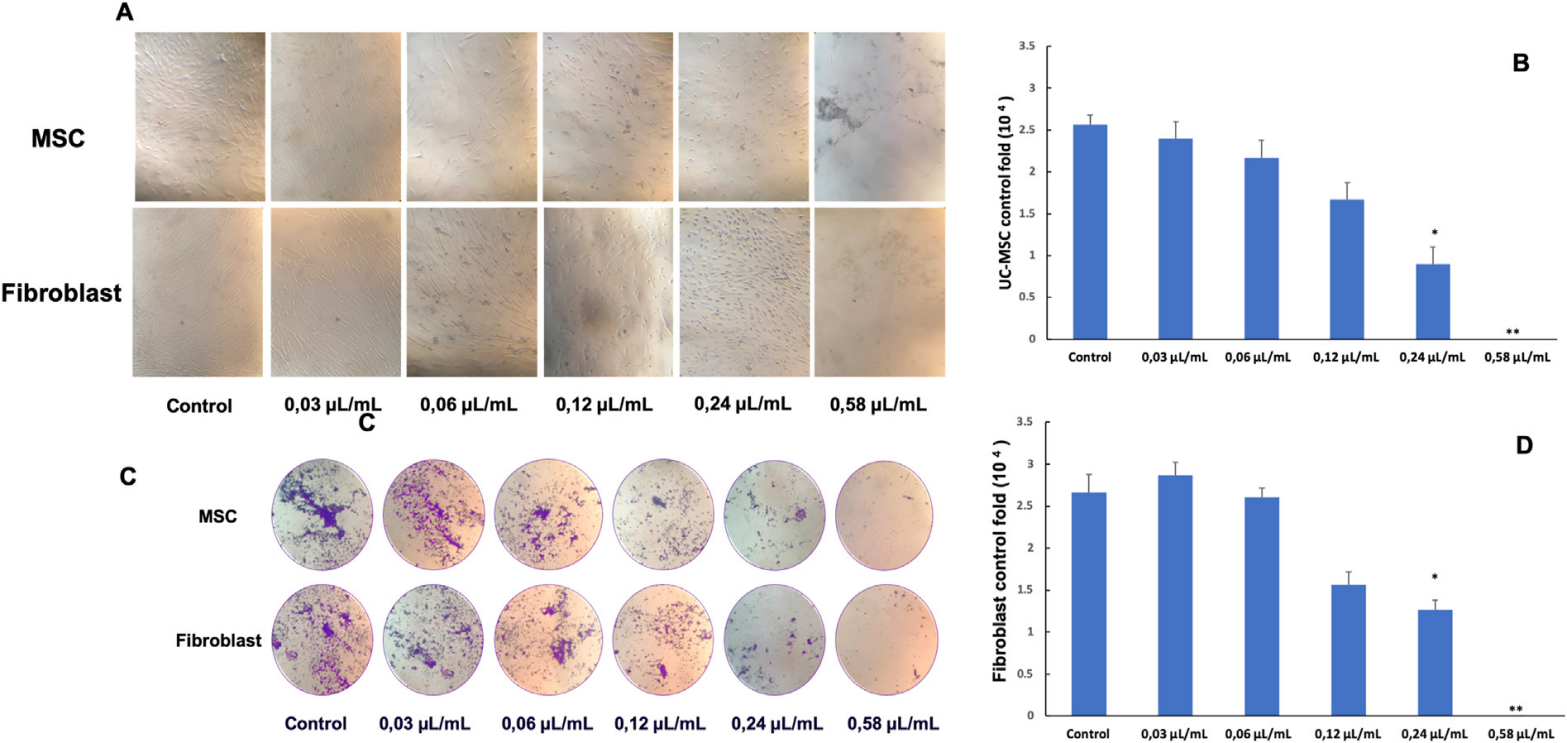
Effect of *Piper betle* L. extracts at different concentrations on UC-MSCs and fibroblast were presented in photos (A,C) and a graph (B,D).

UC-MSCs and ibroblasts were treated with *Piper betle* L. extracts at different concentrations (0.58 μL/mL; 0.24 μL/mL; 0.12 μL/mL; 0.06 μL/mL; 0.03 μL/mL). The results are shown in the image (Figure 4).

Concentration of 0.58 μL/mL, the *Piper betle* L. extract was toxic to all cell lines with more than 90% of dead cells, the UC-MSCs and fibroblast cells were separated into plaque from the stick surface, some of the contractile cells no longer have their characteristic shape (Figure 4A).

The concentration of 0.24 μL/mL; 0.12 μL/mL; 0.06 μL/mL, the cells did not shed at the surface of the T-flash, but the growth rate was slower than the control group, some cells showed shrinkage and after 48 h the cells stopped dividing.

Concentration of 0.03 μL/mL, during the monitoring (48 h) did not detect the phenomenon of cell shrinkage and peeling off the adhesion surface. The cells have the typical shape of fibroblasts, spread evenly on the surface of the bottle, the cells have the same rate of proliferation and development similar to the control group.

After 48 h of adding extract to the culture medium, we carried out dyeing with crystal violet to check for cell death. The results showed that, at the concentration of 0.03 μL/mL extract cells still existed and developed normally. At the concentration of 0.58 μL/mL extract no cells were found to stick to the surface of the T-flash culture bottle (Figure 4C).

### 3.6 *Piper betle* L. extract increased healing rate of scratch wound in UC-MSCs

A scratch wound assay was used to test the impact of *Piper betle* L. extract on the migration of UC-MSCs. UC-MSCs were cultured in DMEM+10%FBS until reaching 80% density proceed to create scratches with a diameter of 800 μm. Then treated with *Piper betle* L. extract at concentration of 0.03 μL/mL for 48 h until the density of cell population higher than 90 %.

**Figure 5:**
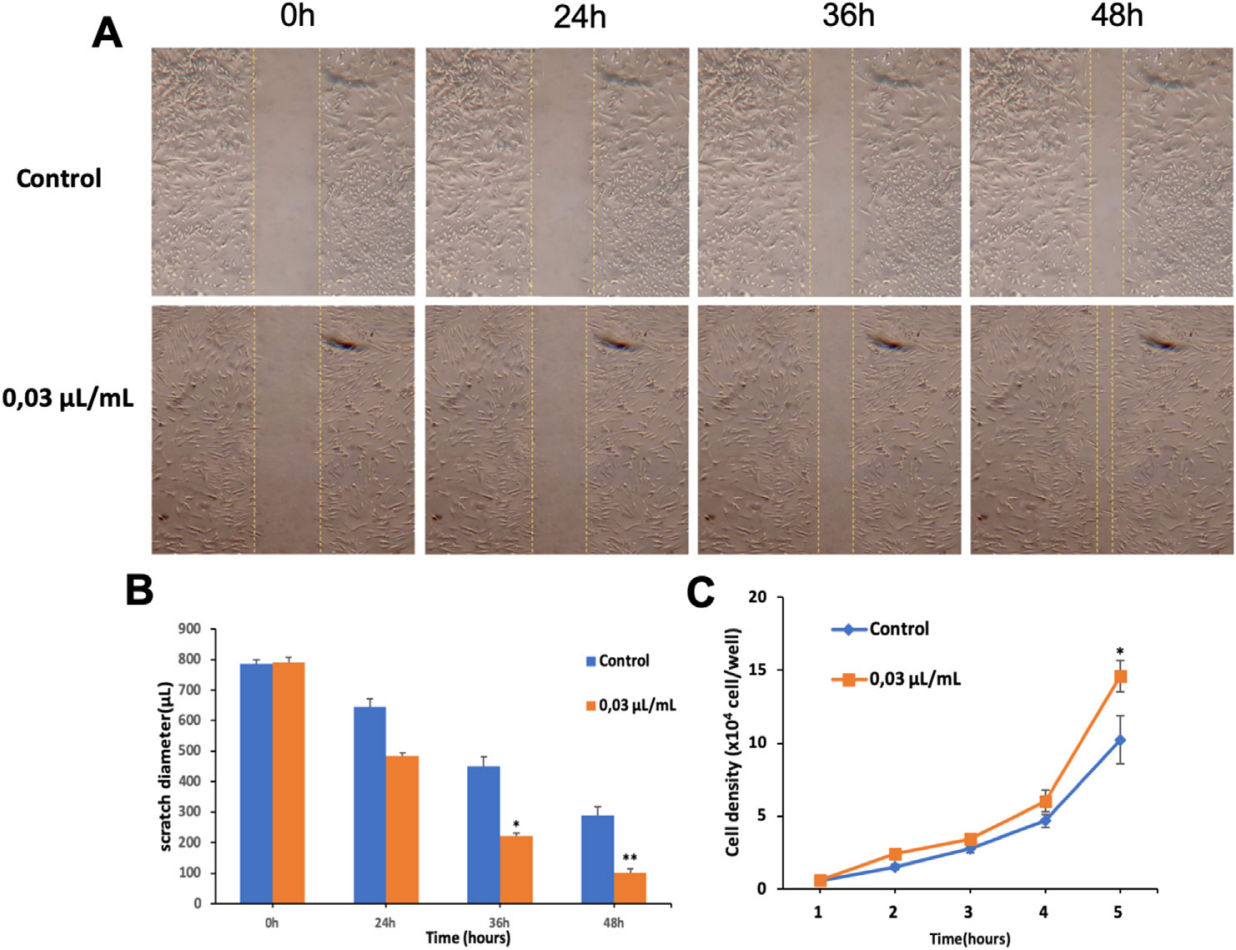
Effect of *Piper betle* L. extract on scratch wound healing of UC-MSCs. (A,B) Scratch diameter is narrowed, (C) Cell density after 5 days.

Scratch diameter in Control group and UC-MSCs group were determined in 0 h (0h), 24 h (24h), 36 h (36h), 48 h (48h) after being formed (Figure 5A, B). In Control group, scratch diameter is narrowed: 138.3 μm; 333.67 μm; 496.67 μm compared to 0h. While Scratch diameter in UC-MSCs group which were treated with *Piper betle* L. extract significantly increased from 24h until 48h: 306 μm; 569.67 μm; 688 μm compared to 0h. Scratch diameter of UC-MSCSS group narrowed 2.21; 1.7; 1.4 -fold compared to the control group. The growth rate of UC-MSCs was supplemented with *Piper betle* L. extract better than the control group and 1.42 times higher (Figure 5C).

### 3.7 *Piper betle* L. extract increased healing rate of scratch wound in fibroblast

We aimed at the healing rate of fibroblast by scratch wound healing assay. When the cell density reaching 80% proceed to create scratches with a diameter of 800 μm. Then treated with *Piper betle* L. extract at the concentration of 0.025 μL/mL for 48 h until the cell population has reached a confluence of more than 90%. Then, within 5 days, the scratch wound healings were tested.

**Figure 6:**
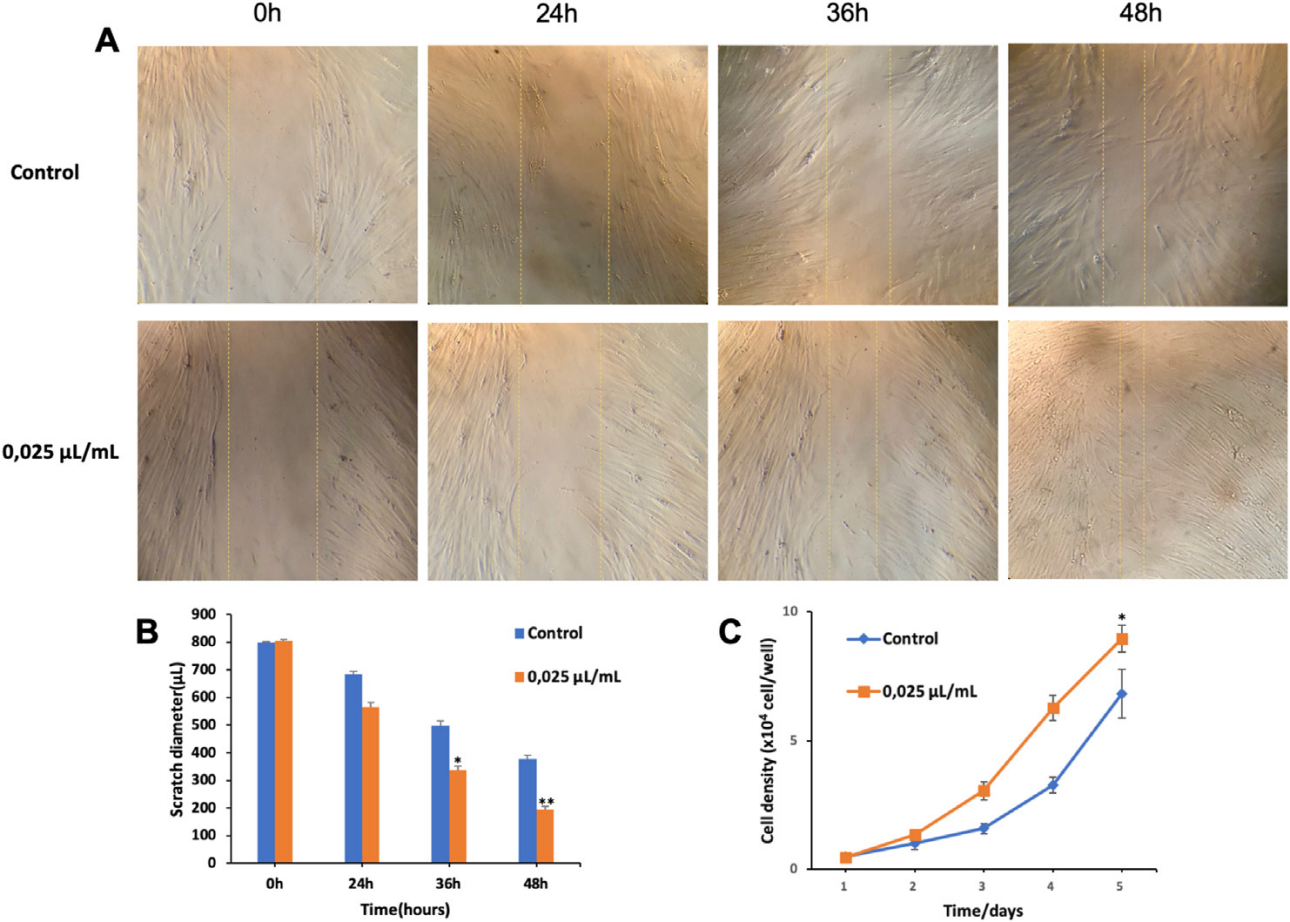
Effect of *Piper betle* L. extract on scratch wound healing of fibroblast. (A,B) Scratch diameter is narrowed, (C) Cell density after 5 days.

Scratch diameter in Control group and UC-MSCs group were determined 24h, 36h, 48h after being formed (Figure 6A, B). In Control group, scratch diameter is narrowed: 115 μm; 299 μm; 420.67 μm compared to 0h. While Scratch diameter in UC-MSCs group which were treated with *Piper betle* L. extract significantly increased from 24h until 48h: 237.67 μm; 467 μm; 610.67 μm compared to 0h. Scratch diameter of UC-MSCs group narrowed 2.05; 1.56; 1.45 -fold compared to the control group. The growth rate of UC-MSCs was supplemented with *Piper betle* L. extract better than the control group and 1.31 times higher (Figure 6C). In the wound healing process, the migration of cells into the wounds plays an important role.

After addition of extract, conduct staining of fibroblasts and check FACS level of expression of specific markers CD90, α-SMA, CD34, CD45.

**Figure 7:**
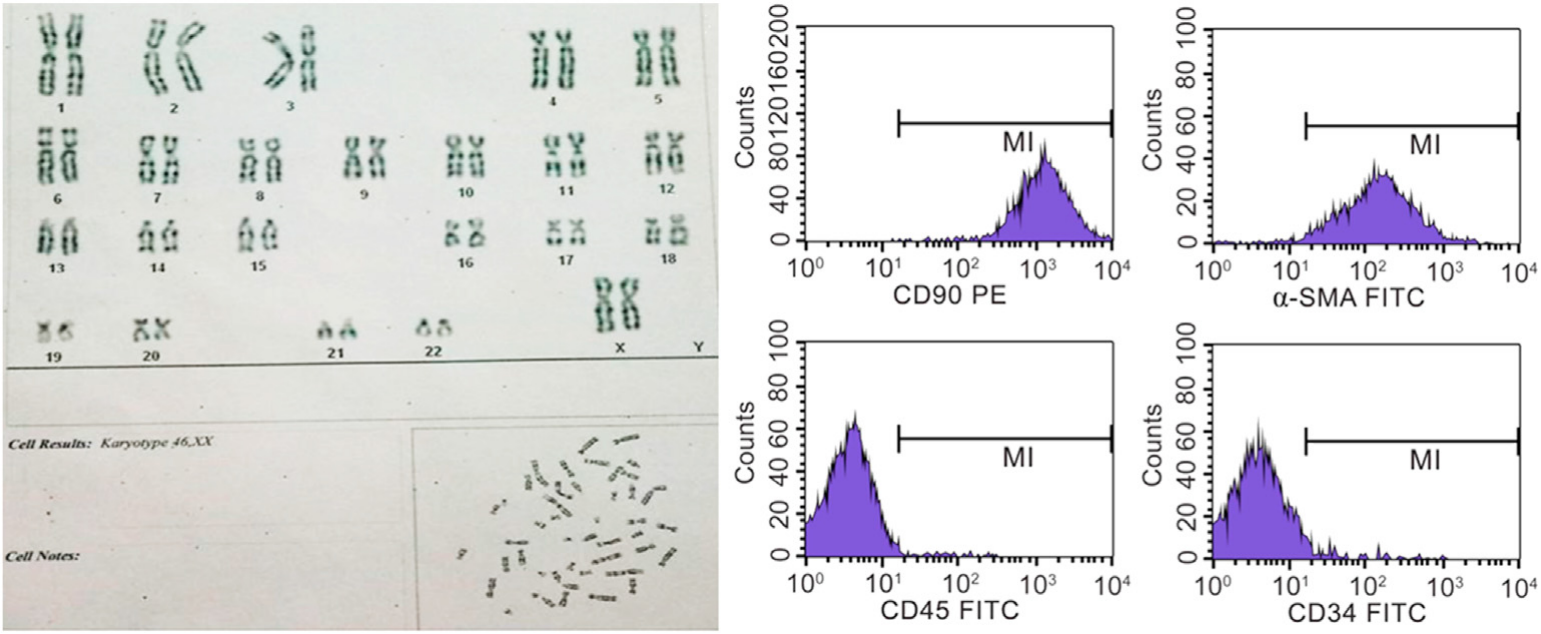
Chromosome analysis and expression of the CD 90, α-SMA, CD34, CD45.

The result is shown in Figure 7. The analyzed image showed no mutations in the number and structure of chromosomes.FACS results showed fibroblasts after supplementing with *Piper betle* L. extract: positive for CD90 and α-SMA, negative for CD34 and CD45, proving that fibroblasts maintained stable chromosome structure and featured marker when incubated with *Piper betle* L. extract.

### 3.8 Express fibroblast activation markers

**Figure 8:**
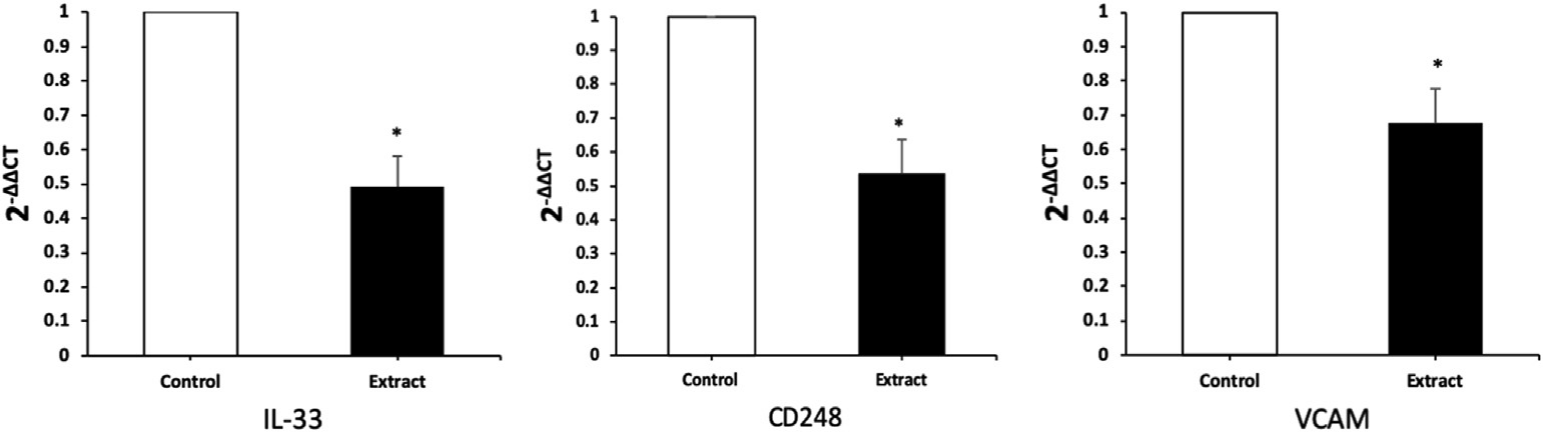
Percentage of cells positively stained for samples for expression of CD248, IL-33, VCAM after addition of *Piper betle* L. extract (∗p < 0.05 (Students t-test)).

The activation markers were substantially greater in expression CD248, IL-33, VCAM. in the control group compared with the extract group (p < 0.05) (Figure 8). In the scratch model, after addition of extract, all factors showed a decrease. Analysis of expression revealed that IL-33 was most reduction with approximately 50,8% of extract group versus in control group (Figure 8) followed by CD248 (46.13%, p < 0.05) and VCAM (32.1%, p < 0.05).

## 4 Discussion

In this work, the reason for using fibroblasts and MSC to study betel leaf extract is that they are strongly associated. While fibroblast can be as the primary cell type in healing wound process on the grounds some characteristics of them like migration and proliferation at sites of injury when appearing the release of growth factors, synthesize some cytokines and extracellular matrix-associated macromolecules, including collagen and fibronectin (FN) [2,3,4]; MSCs perform their role such as: providing a possibility to compensate for defective fibroblasts in chronic skin wounds. Therefore, MSCs might be a potential source of fibroblast differentiation through using some connective tissue growth factors, moreover, MSCs also have some their advantage like be as unlimited cell sources, can tackle the obstacle of currently existing therapy for ulcers [25]. In addition, Denise R *et al.* (2018) showed that the conditioned media from MSC stimulated cell migration of HDFs in vitro by 48% [26]. This suggests that finding an element or herb that can facilitate the proliferation of fibroblasts and MSC will greatly assist in wound healing.

*Piper betle* L. is a traditional herbal medicinal and used in many traditional treatments as also as the health improvement effect in Vietnam. Traditionally, the positive effects of betle leaves and their derivatives have been exploited to treat a variety of open wound diseases like cuts, injuries, inflammations, cold cough, indigestion, halitosis, boils and abscesses, headache, mastoiditis, leucorrhea, abrasion [27,28].

Our research results for *Piper betle* L. extract on *in vitro* scratch healing model showed the effect of promoting the wound healing process. This has been shown by a large rise in the rate of scratch decrease, ANOVA analysis results and images showing that scratch healing depends on the concentration of the extract. At a concentration of 0.058 μL/mL, the results showed that the extract was toxic to UC-MSCs and fibroblast cell lines, the cells were no longer able to survive. However, the more diluted concentrations, the *Piper betle* L. extract, show a positive signal. Studies on scratch models and growth curves showed that for UC-MSCs, the concentration of 0.03 μL/mL positively affected cell proliferation. After 48 h of observation, the size of the scratches was narrowed to 688 μm and 1.4 times that of the control group. Meanwhile, for fibroblast, the concentration of 0.025 μL/mL has the best influence on the proliferation process. The diameter of the scratches has shrunk to 610 μm, 1.45 times that of the control group.

In this study, We have demonstrated that *Piper betle* L. extract has to reduce the presence of IL-33, CD248, VCAM so that it can activate the process of proliferation in fibroblast. CD248 is a transmembrane receptor with ligands that include collagen 1 and are upregulated by inflammation in inflammatory reactions; recent studies indicate that overexpression of CD248 can be differentiated into tumor stromal fibroblasts [7]. CD248 expression levels were closely associated with tumor grade, invasiveness, and poor prognosis in breast cancer and neuroblastoma [29,30]. VCAM-1 is a surface glycoprotein that facilitates leukocyte adhesion and subsequent recruitment [31] and similarly the cytokines interleukin 33 (IL-33) as an alarmin in early and recruitment of circulating immune cells IL-1β, TNFα, IL-4, IL-6 [6]. Overexpression of markers of fibroblast leads to prolonged inflammatory processes therefore suggesting this may drive disease chronicity which is often associated with oxidative stress and triggers negative responses to structure and cell repair. *Piper betle* L. extracts contain phenolic components that have been shown to reduce reactive oxygen species (ROS) including by inhibiting lipid peroxidation [32,33,34,35]. Our research shows the relationship between the extract and the factors IL-33, CD248, VCAM which act as intermediates to help *Piper betle* L. extract regulate the negative effects on cells, thereby reducing stressoxydase and speeding up the process of wound healing. Lien *et al.* (2015) by scratch wound model showed *Piper betle* L. extract stimulating proliferation for NIH3T3 cell lines [33]. The extract acting as antioxidant agents reducing the content of Malondialdehyd (MDA) which can cause tissue damage and DNA damage. When research on the molecular level, Lina *et al.* (2017) showed that *Piper betle* L. extract regulates the expression of some genes which relate to senescence in replicative senescent Human Diploid Fibroblasts (HDFs) [16]. Their findings revealed that Piper Betle L. Extracts could increase the proliferation of cells in young (143%), presenescent (127.3%), and senescent (157.3%) HDFs. For UC-MSCs, currently there is very little research on the application of *Piper betle* L. to affect umbilical cord stem cells. Our research shows that betel leaf extract at the appropriate concentration has a positive effect on the ability of the cell to stimulate proliferation. *Piper betle* L. extracts at different concentrations have the ability to stimulate proliferation to many different cell lines. However, these are initial studies on scratch healing models, in-depth studies are needed to determine the mechanism of influence of *Piper betle* L. extract. In our preliminary study providing additional information on the potential effect of *Piper betle* L. extract on the proliferation of UC-MSCs and, showing the potential of *Piper betle* L. extract in regenerative medicine, ability to homogenize with many different cell lines, helping to accelerate wound healing process.

These results indicate the potential of *Piper betle* L. extract in speeding up the healing of the scratch wound. We can use the extract with the appropriate concentration to serve the wound healing activities and identify a key component of the extract's wound healing process to develop an optimal clinical regimen by changing the concentration or combining it with cell lines or other drugs.

## 5 Conclusions

- *Piper betle* L. extract collected in Hue city, Thua Thien Hue province contain 16 different compounds (GC-MS result), of which ingredients such as eugenol, phytol have been shown to have antibacterial, antifungal antioxidant properties cell and anti-cancer.
- In this preliminary study, we concluded that *Piper betle* L. extract at concentrations of 0.025 μL/mL stimulated proliferation of fibroblasts and 0.03 μL/mL stimulated proliferation of UC-MSCs, thereby promoting wound healing on in vitro models.
- Research also showed that the extract had a negative effect on the cell lines, toxic at a concentration of 0.58 μL/mL.
- *Piper betle* L. extract has the effect of reducing the presence of oxidative stress factors such as IL-33, CD248, and VCAM when cells are damaged, so it can it can speed up the healing process.
- Our results show that natural extracts of *Piper betle* L. with phenolic compounds have great potential for regenerative medicine applications by reducing the expression of inflammatory factors and oxidative stress. (IL-33, CD248, VCAM). In the future, more in-depth studies will explain the mechanism of the use of *Piper betle* in the treatment of diabetes, conjunctivitis, mastitis, burns, ulcers, antibacterial, anti-inflammatory, antioxidant, anti-inflammatory, and cancer. This use of *Piper betle* L. extract paves the novel way, alternative systems, and combination to treat skin wounds effectively.

## Declarations

### Author contribution statement

Chế Thị Cẩm Hà: Conceived and designed the experiments; Performed the experiments; Analyzed and interpreted the data; Contributed reagents, materials, analysis tools or data; Wrote the paper.

Lê Hoàng Duy Minh: Performed the experiments; Wrote the paper.

Nguyễn Hữu Đạtt: Analyzed and interpreted the data.

### Funding statement

This work was supported by the Domestic Master/PhD Scholarship Programme of Vingroup Innovation Foundation, Vietnam.

### Data availability statement

Data included in article/supplementary material/referenced in article.

### Declaration of interests statement

The authors declare no conflict of interest.

### Additional information

No additional information is available for this paper.
